# Characterization of the Exopolysaccharide Produced by *Salipiger mucosus* A3^T^, a Halophilic Species Belonging to the *Alphaproteobacteria*, Isolated on the Spanish Mediterranean Seaboard

**DOI:** 10.3390/md8082240

**Published:** 2010-07-30

**Authors:** Inmaculada Llamas, Juan Antonio Mata, Richard Tallon, Philippe Bressollier, María C. Urdaci, Emilia Quesada, Victoria Béjar

**Affiliations:** 1 Microbial Exopolysaccharide Research Group, Department of Microbiology, Faculty of Pharmacy, Cartuja Campus, University of Granada, 18071 Granada, Spain; E-Mails: illamas@ugr.es (I.L.); jonanmg@gmail.com (J.A.M.); equesada@ugr.es (E.Q.); 2 IUT, Département Génie Biologique, allée André Maurois, 87065 Limoges, France; E-Mails: richard_tallon@yahoo.fr (R.T.); philippe.bressollier@unilim.fr (P.B.); 3 Laboratorie de Microbiologie et Biochimie Appliquée, ENITA de Bordeaux, 1 Cours du Général de Gaulle, BP 201, 33175 Gradignan, France; E-Mail: m-urdaci@enitab.fr

**Keywords:** exopolysaccharides, Salipiger mucosus, halophilic bacteria, fucose, sulfates

## Abstract

We have studied the exopolysaccharide produced by the type strain of *Salipiger mucosus*, a species of halophilic, EPS-producing (exopolysaccharide-producing) bacterium belonging to the *Alphaproteobacteria*. The strain, isolated on the Mediterranean seaboard, produced a polysaccharide, mainly during its exponential growth phase but also to a lesser extent during the stationary phase. Culture parameters influenced bacterial growth and EPS production. Yield was always directly related to the quantity of biomass in the culture. The polymer is a heteropolysaccharide with a molecular mass of 250 kDa and its components are glucose (19.7%, w/w), mannose (34%, w/w), galactose (32.9%, w/w) and fucose (13.4%, w/w). Fucose and fucose-rich oligosaccharides have applications in the fields of medicine and cosmetics. The chemical or enzymatic hydrolysis of fucose-rich polysaccharides offers a new efficient way to process fucose. The exopolysaccharide in question produces a solution of very low viscosity that shows pseudoplastic behavior and emulsifying activity on several hydrophobic substrates. It also has a high capacity for binding cations and incorporating considerable quantities of sulfates, this latter feature being very unusual in bacterial polysaccharides.

## 1. Introduction

Microbial exopolysaccharides (EPSs) are polymers that consist principally of carbohydrates and are excreted by some bacteria and fungi onto the outside of their cell walls. Their composition and structure is very varied: they may be either homo- or heteropolysaccharides and may also contain a number of different organic and inorganic constituents [1]. Microbial EPSs occur naturally in many habitats. They are important in the formation of biofilm, a structure involved in the adherence to surfaces and in the protection of bacteria against noxious influences of the environment. They can be readily prepared in the laboratory by fermentation. Increasing attention is being paid to these molecules because of their bioactive role and their extensive range of potential applications in pharmaceuticals as antiangiogenic [2] or antiviral agents [3], and in agriculture and various other industrial areas [4].

The advantages of microbial polysaccharides over plant or marine macroalgal polymers are their novel functionality, easily reproducible chemical and physical properties and stable cost and supply [5]. During the past 50 years a considerable number of EPSs have been described, but few have achieved great commercial success, due either to their being unable to offer better properties than those already on the market or to difficulties in finding new practical applications [4].

A new approach to encountering EPSs with novel properties might entail investigating different environments such as hypersaline habitats, since most of the strains used so far for the industrial production of EPSs belong to a small number of taxa, which are generally non-halophilic, soil-dwelling diazotrophs and often plant-associated. Because of the extreme nature of hypersaline environments they may feasibly harbor unusual microorganisms of biotechnological interest and so for the last few years we have been carrying out a wide research program, looking at microorganisms living in habitats such as these in an attempt to find new EPSs with different characteristics. This has resulted in our describing the EPSs synthesized by several moderately halophilic species published by our group, belonging to the *γ-Proteobacteria: Halomonas eurihalina* [6], *H. maura* [7], *H. ventosae* [8], *H. anticariensis* [9], *Alteromonas hispanica* [10] and *Idiomarina ramblicola* and *I. fontislapidosi* [11]. In general, the polymers produced by these bacteria show potential interest as viscosifying, jellifying and emulsifying agents and as metal-binding compounds [12–14]. The most interesting polymers obtained so far from halophilic bacteria are mauran, produced by *H. maura*, which has a viscosifying activity similar to that of xanthan gum [15,16], and polymers from *H. eurihalina* species, which have emulsifying activity and jellifying properties at acidic pH [17–23].

We describe here an EPS produced by the type strain (A3^T^) of *Salipiger mucosus* [24], a halophilic bacterium belonging to the *Alphaproteobacteria* class. To our knowledge, this is the first time a halophilic *Alphaproteobacteria* has been found to produce an EPS. To understand better the characteristics of EPSs and thus be able to apply them successfully to biotechnological ends it is essential to optimize their production and to identify their components and physical-chemical properties.

## 2. Results

### 2.1. Kinetics of Growth and EPS Production

**Figure 1:**
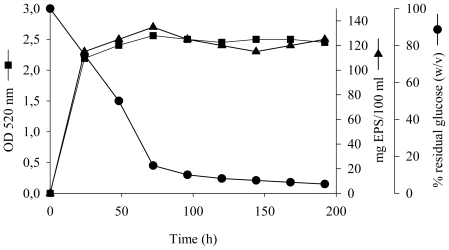
Growth profile and EPS production by *S. mucosus* strain A3^T^ in MY medium at 7.5% total salts *versus* consumption of glucose. 100% of residual glucose corresponds to 10 g/L of glucose.

To study the synthesis of the EPS as a function of the growth phase, *S. mucosus* strain A3^T^ was grown in MY medium containing 7.5% (w/v) total salts and an initial glucose concentration of 10 g/L for eight days at 32 °C and 100 rpm without control over pH, as shown in Figure 1. Glucose metabolism led to an increase in biomass (final OD_520_ of 2.6) and an EPS accumulation of 1.35 g/L (0.1 g of EPS per gram of dry cell weight). The kinetics of EPS production by *S. mucosus* strain A3^T^ showed that it was excreted mainly during the exponential growth phase but continued to a lesser extent during the stationary phase. Production ceased when residual glucose was about 1.5 g/L. The quantity of EPS in the culture decreased considerably after 72 hours of incubation.

### 2.2. Influence of Culture Parameters on Growth and EPS Synthesis

We studied the influence of different cultural parameters in order to be able to improve EPS production by *S. mucosus* strain A3^T^. Yield was always directly related to the quantity of biomass in the culture. A sea-salt concentration of 2.5% (w/v) proved best in terms of production. One noteworthy result was that the bacterium grew and produced EPS with all the carbon sources assayed. Growth remained unimpeded when glucose was increased to above 1% (w/v) in the culture but without any significant increase in synthesis. As far as incubation temperature is concerned, yields were substantially lower at both 22 °C and 42 °C than at 32 °C, concomitant with a significant inhibition in bacterial growth. Similar results were obtained when the pH was lower or higher than 7.0. In the same way, both static incubation and a stirring rate of more than 100 rpm gave similarly lower growth and lower EPS yields.

### 2.3. Electron Microscopy

**Figure 2:**
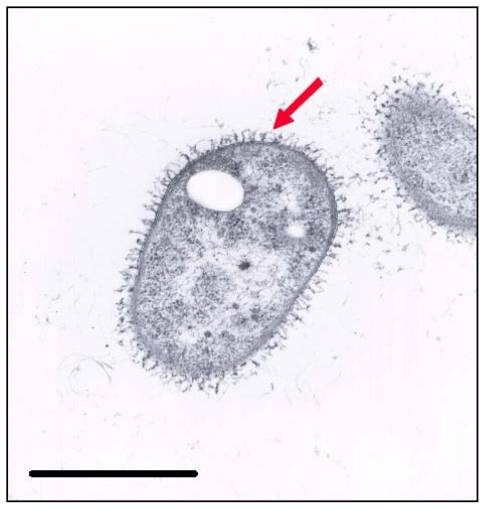
Transmission electronic microscopy photograph of *S. mucosus* strain A3^T^ stained with the specific stain for polysaccharide ruthenium red. Bar: 1 μm. Arrow indicates the EPS of the strain.

Figure 2 shows the appearance of strain A3^T^ after 24 hours incubation. It is noteworthy that during the early stage of incubation the EPS was in close contact with the cell, but afterwards it was diffused fairly evenly throughout the medium as an amorphous slime with no indication of any higher concentrations near the cell wall (data no shown). This phenomenon explains why we were able to extract higher quantities of EPS from the third day onwards.

### 2.4. Chemical Composition

When collected under optimum conditions the EPS produced by *S. mucosus* strain A3^T^ was composed of 53.1% (w/w) carbohydrates, 1.6% (w/w) proteins, 0.9% (w/w) acetyls, 0.9% (w/w) sulfates, 0.1% (w/w) phosphates, 1.9% (w/w) hexosamines and 6% (w/w) pyruvic acid; each value representing the average of three measurements. The EPS collected under other conditions assayed had the same basic composition but the carbohydrate content was always lower (data not shown). The samples obtained under optimum conditions were purified by anion exchange chromatography (AEC). A single major peak of sugar-bearing material was eluted from the column with NaCl concentrations of about 1 M, indicating a single acidic EPS. The components of the carbohydrate fraction of the EPS from *S. mucosus* strain A3^T^ were (%, w/w): 19.7 glucose, 34 mannose, 32.9 galactose and 13.4 fucose; each value representing the average of three measurements.

### 2.5. Physical Properties

**Figure 3:**
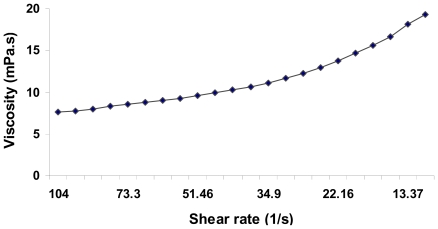
Viscosity of a 0.5% (w/v) solution of EPSs produced by *S. mucosus* A3^T^ in MY medium at 2.5% total salts.

The average molecular mass of the EPS produced by *S. mucosus* strain A3^T^ extracted after three days from MY medium with 2.5% (w/v) salts was 250 kDa. To evaluate the rheological properties of the EPS we measured the viscosity of an aqueous solution with 0.5% (w/v) of EPS over a range of different shear rates. The flow curve in Figure 3 shows the pseudoplastic character of a solution of the polymer, its viscosity decreasing concomitantly with shear rate. The viscosity of the solution was quite low.

The emulsifying activity of the EPS is shown in Table 1. It was capable of stabilizing different mixtures of oil and water in which the hydrophobic phase was either a hydrocarbon or a vegetable or mineral oil; the polymer’s activity was more efficient than the chemical surfactants (Triton X-100 and Tween 80) used as controls when tetradecane, octane, kerosene and xylene were used as substrates. The highest emulsifying activity was obtained with crude oil (higher than with xanthan). As far as metal chelation is concerned, it chelated 15.7, 43.5 and 8.7 mg of copper, lead and cobalt, respectively, per gram of EPS.

## 3. Discussion

We report here, the first isolation and characterization of an exopolysaccharide produced by a halophilic bacterium belonging to the *Alphaproteobacteria* class. *Salipiger mucosus* A3^T^ excretes significant quantities of EPS when cultivated under optimum growth conditions, that is to say, the conditions that result in the highest speed of cell division and ultimately the greatest quantity of bacterial growth. These results do not agree with those of some other authors, who maintain that cell growth and EPS formation usually have different nutritional requirements [25,26]. The different culture conditions assayed did not change meaningfully the chemical composition of the EPS, although production decreased significantly when they were unfavorable (data not shown). EPS production by *S. mucosus* strain A3^T^ exhibited a fermentation kinetic similar to that of mauran [15] and those produced by *H. ventosae* and *H. anticariensis* [13] and *Alteromonas hispanica* and *Idiomarina fontislapidosi* [14]. It began early, during the exponential growth phase, then increased concomitantly with the rise in number of viable cells, only ceasing when the glucose substrate was almost completely consumed. The highest quantity of EPS was obtained after a period of three days. After the optimum incubation time there was a decrease in the quantity of EPS in the culture, which may well be due to enzymatic degradation, as has also been reported with other EPSs [27].

With regard to the chemical composition of our EPS, its sulfate content, together with the presence of phosphates and fucose, are especially interesting. Sulfates are not commonly found in microbial EPSs, although they are present in all the EPSs produced by the halophilic bacteria described by our group [see for example 13–15,18] and also in many marine bacteria and in cell wall polysaccharides from red and brown macroalgae. Sulfated EPSs are of great potential interest in medicine since they have a number of bioactive properties: anticoagulant, antiangiogenic, antiproliferative, antiviral, *etc.* [2,3,28]. Phosphate groups, which have also been observed in other EPSs [13,29], could confer important properties on them because they are essential to the activation of lymphocytes [30] and in some antitumoral processes [31]. Fucose and fucose-rich oligosaccharides can be used in biocosmetics, in the field of medicine and in the food industry [32]. The polymer from *S. mucosus* strain A3^T^ may prove to be a simple source of fucose, as reported for the EPSs excreted by *Klebsiella pneumoniae* and *Clavibacter michiganensis* [32] since chemical synthesis or extraction from algae is laborious and expensive and they are often in short supply.

One important feature of the emulsions produced by the EPS described here is that they are very stable and are composed of small, uniform droplets, resulting in a fine, smooth consistency. Other known polysaccharides, such as xanthan, are capable of producing stable emulsions but they tend to be thicker and more viscous, which is not very desirable for some of the uses for which emulsifiers such as these are intended [33]. Proteins play an important role in the emulsifying capacity of some exopolysaccharides [13,34]. The EPS from our halophilic strain contains protein concentrations of 1.6% (w/w). In the same way, emulsan, a biosurfactant obtained from *Acinetobacter calcoaceticus*, is a complex of polysaccharides and proteins that emulsifies mixtures of water and aliphatic, aromatic or cyclic hydrocarbons but cannot emulsify pure hydrocarbons [35]. *Salipiger mucosus* strain A3^T^, on the other hand, produces an EPS capable of emulsifying higher percentages of pure hydrocarbons (tetradecane, octane, kerosene, xylene and crude oil) than the chemical surfactants used in comparison, and where crude oil is concerned, higher even than xanthan. Apart from this, the presence of acetyl groups renders the EPS somewhat hydrophobic, which might contribute to its emulsifying capacity, as Ashtaputre *et al*. [36] have described for the EPS produced by *S. paucimobilis*.

EPSs with high concentrations of charged components often form gels in the presence of metal ions and have great potential for removing toxic metals from polluted environments and wastewater as an alternative to other more aggressive methods [37]. EPS solutions from *S. mucosus* strain A3^T^ did not produce stable gels in the presence of the salts we tested, but they did chelate several metals with considerable efficiency. Anionic EPSs generally prefer to bind cations with large ionic radii [38], which agree with our findings, since our polysaccharide showed a high capacity to bind lead. It may well be that acetyls bring more electron-donating groups into the vicinity of the binding site, thus allowing the larger Pb ions to bind more firmly [38]. Although we are not yet sure of the actual mechanism involved when ions bind to these polymers, this type of chelation could be classified as biosorption, as mentioned by Valls and de Lorenzo [39]. Whatever the case may be, the strong chelating property of this polymer offers the possibility of its being used as a biosorbent in the treatment of polluted water and other such environments.

## 4. Experimental

### 4.1. Bacterial Strain

We used *Salipiger mucosus* strain A3^T^ (=CECT 5855^T^), described by our group after an extensive program of isolating halophilic EPS producers from 19 hypersaline environments in Spain and Morocco [40]. *S. mucosus* strain A3^T^ was isolated from a hypersaline soil located in a solar saltern in Calbanche (Murcia) on the Spanish Mediterranean seaboard.

### 4.2. Optimization and Analysis of the Kinetics of EPS Production

The bacterial strain was cultivated in MY medium [41] supplemented with 7.5% (w/v) salts [42].

The EPS was isolated using the method described by Quesada *et al.* [23]. Briefly, the culture was centrifuged and the supernatant precipitated with cold ethanol before being ultracentrifuged, dialyzed against distilled water and lyophilized [43].

To establish which conditions lead to optimum EPS production, we assayed the following variables: incubation time (1–8 days), incubation temperature (22, 32 and 42 °C), sea-salt concentration (1, 2.5, 5, 7.5, 10, 15, and 20% (w/v)), carbon source (glucose, sucrose, mannose, galactose), glucose concentration (0, 1, 2, 5, 7 and 10% (w/v)) and incubation either in a rotating shaker (100 and 200 rpm) or static conditions.

Microbial growth and EPS production were monitored in batch cultures in 500-mL Erlenmeyer flasks containing 100 mL medium (three replicate flasks per experiment). Bacterial growth was determined by measuring optical density at 520 nm. Any residual carbon source was calculated using the glucose-oxidase technique [44].

### 4.3. Electron Microscopy

Ultrathin sections of bacterial cells were negatively stained as described elsewhere [7].

### 4.4. Chemical Analysis

Total carbohydrates [45], proteins [46], acetyls [47], pyruvate [48], hexosamines [49], sulfates and phosphates [13] were analyzed.

The EPS was purified and its negative net charge analyzed by AEC on a 1.5 m × 20 cm quaternary methyl ammonium Accel Plus column (Waters) eluted at a flow rate of 2 mL/min with 0.05 M NH_4_HCO_3_ (pH 8.0), followed by a linear gradient of 0.05 to 2 M NaCl in the same buffer. It was also monitored by UV detection at 210 nm. Five milliliters fractions were collected to determine their sugar composition and molecular mass.

Sugar composition was determined as described by Chaplin [50]. The purified EPS (100 μg) was subject to methanolysis with methanolic HCl (0.9 M) for 16 h at 80 °C. The resulting mixture of methylglycosides was dried under nitrogen at room temperature and re-N-acetylated by the addition of 50 μL dry methanol, 5 μL pyridine and 5 μL acetic anhydride, dried under nitrogen and then derivated with 15 μL trimethylsilylimidazole (Alltech) at room temperature for 30 min. The re-*N*-acetylated trimethylsilylated glycosides were analyzed on a BP1 fused-silica capillary column (12 m × 0.32 mm, SGE) with a Peri 2000 GLC-FID chromatograph (Perichrom) using a temperature program of 140–240 °C at 6.1 °C/min followed by isothermal elution.

### 4.5. Physical Properties

Apparent molecular mass was determined by high-performance size-exclusion chromatography (HPSEC) done on a 600 E system (Waters) equipped with a PL aquagel-OH 60, 8 μm column (30 cm × 7.5 mm) (Polymer Laboratories), eluted with a 0.2 M sodium-acetate buffer (pH 5.1) at a flow rate of 0.8 mL/min. The sample volume was 20 μL, containing 25 μg of EPS. Compounds were detected using refractive-index monitoring (Model 475, Kontron Instruments) and dextrans (7 × 10^4^ Da to 4.9 × 10^6^ Da, Sigma) were used as standards.

For rheological analysis the EPS was dissolved in distilled water (0.5%, w/v) and measurements were made at 25 °C in a controlled-stress Bohlin CSR10 rheometer.

The emulsifying activity of the EPS was determined by a modified version of the procedure described by Cooper and Goldenberg [51]. Equal volumes of the different EPS solutions (0.5%, w/v) in distilled water and various hydrophobic substrates were added to 105 × 15 mm glass tubes. The mixtures were shaken vigorously using a vortex and allowed to stand for 24 h at 4 °C. Emulsifying activity was expressed as the percentage of the total height occupied by the emulsion. The hydrophobic substrates were sunflower and olive oils (commercial brands), mineral oil, tetradecane, octane, kerosene, isopropyl myristate, xylene, toluene, vaseline oil, hexadecane (all from Sigma), and petrol, diesel and crude oil. Tween 80 and Triton X-100 (from Sigma) and xanthan were used as control surfactants. The emulsions were observed under a light microscope to determine their size and the uniformity of the drops in the oil phase and whether or not there was creaming or flocculation.

Metal-binding analyses were made as described by Geddie and Sutherland [38]. The EPS was applied to an Amberlite IR 120H^+^ cation exchange column (Avocado) buffered with doubly distilled water to convert it into the acidic form. 5 mL polysaccharide solutions (0.5%, w/v) were put into dialysis tubing in flasks containing 200 mL of each appropriate metal-salt solution and shaken at 100 rpm for 24 h at 30 °C. The quantity of metal removed from the solution, *i.e.*, bound to the polymer, was calculated by measuring the ions in solution at 0 h and those remaining after 24 h by atomic absorption spectrophotometry. Controls were made by placing 5 mL distilled water in dialysis tubing with the various metal-salt solutions. The metal salts used were cupric sulfate, cobalt chloride and lead acetate (Sigma).

## 5. Conclusions

Few EPSs with properties of interest to biotechnology have been described during the last decade. Among these few, however, we include the EPS produced by *Salipiger mucosus* strain A3^T^, the first halophilic, EPS-producing bacterium discovered belonging to the *Alphaproteobacteria*. The most remarkable property of the EPS in question is its unusual composition—with a high fucose content—and therefore it offers possible novel applications as a biological agent. We are currently studying its antitumoral activity and also its use as a source of fucose and fucose-oligosaccharides.

## Figures and Tables

**Table 1:** Emulsifying activities of the EPS produced by *S. mucosus* strain A3^T^ *.

	*S. mucosus* A3^T^	Triton X-100	Tween 80	Xanthan gum
Sunflower oil	70 ± 1.41	62.5 ± 1.82	62 ± 1.67	88.95 ± 1.60
Mineral oil	71 ± 1.60	67.5 ± 1.41	70 ± 1.15	90.3 ± 1.43
Olive oil	60.3 ± 1.52	60 ± 1.52	62.5 ± 1.64	100 ± 1.94
Tetradecane	75 ± 2.08	62.5 ± 1.82	62.5 ± 1.10	90.3 ± 0.98
Octane	70 ± 1.51	60 ± 1.52	60 ± 0.70	93.3 ± 2.18
Kerosene	70 ± 0.51	62.1 ± 2.12	60 ± 1.40	89 ± 1.02
Isopropyl myristate	67.5 ± 0.70	67.5 ± 0.70	67.5 ± 1.02	100 ± 1.34
Petrol	20 ± 3.01	70 ± 1.52	32.5 ± 2.60	92.5 ± 2.45
Diesel	37.5 ± 0.70	62.5 ± 2.12	65 ± 1.70	87.5 ± 3.01
Crude oil	95 ± 0.57	60 ± 0.70	60 ± 0.60	89.75 ± 1.32
Xylene	47.5 ± 0.70	12.5 ± 1.52	12.5 ± 1.30	86.5 ± 1.78
Toluene	52.6 ± 3.53	12.5 ± 0.70	60 ± 1.80	100 ± 1.75
Vaseline oil	52.6 ± 2.50	65 ± 0.57	60 ± 2.55	100 ± 1.67
Hexane	50 ± 2.51	50 ± 1.52	42 ± 0.66	100 ± 2.25

* Expressed as the percentage of the total height occupied by the oil-water emulsion after 24 h; 0.5% (w/v) EPS or chemical surfactant was used as emulsifier. Each value represents the average of three measurements.
